# Hydrogen Carrier Gas Method Translation in Comprehensive Two-Dimensional Gas Chromatography for Sustainable Nontargeted Analysis

**DOI:** 10.1021/acs.analchem.6c03350

**Published:** 2026-07-17

**Authors:** Kira M. Fisher, Emma L. Macturk, Katelynn A. Perrault Uptmor

**Affiliations:** Nontargeted Separations Laboratory, Chemistry Department, William & Mary, Integrated Science Center 1053, 540 Landrum Drive, Williamsburg, Virginia 23188, United States

## Abstract

The selection and control of carrier gas are critical factors influencing the performance of both one-dimensional gas chromatography (1D GC) and comprehensive two-dimensional gas chromatography (GC×GC). Helium is widely used due to its inertness, but increasing cost, limited supply, and reliance on fossil fuel extraction raise sustainability concerns. Hydrogen offers a viable alternative, with faster optimal linear velocity to reach comparable efficiency, and more sustainable production through generators relying on water electrolysis. While tools exist to translate 1D GC methods from helium to hydrogen, their application to GC×GC has not been well established, particularly for parameters unique to multidimensional separations. This work presents the translation of helium to hydrogen carrier gas for cryogenically modulated GC×GC-MS. Three translated methods derived from 1D tools were evaluated alongside modulation period using standard mixtures, followed by validation with authentic fingermark residue samples in a forensic application. The translate option, using a combination of flow and temperature adjustments that matched column efficiency between helium and hydrogen, delivered comparable peak capacity, as well as acceptable S/N, tailing factors, and resolution values between key peak pairs. Modulation period could be reduced by 50%, providing a practical guideline for method translation. Hydrogen reduced overall run time by up to 60%, increasing sample throughput and lowering per-sample energy consumption. Although sustainability comparisons are nuanced, hydrogen showed improved green chemistry alignment overall, supported by higher sustainability metric scores and gains in efficiency and resource use.

## Graphical abstract

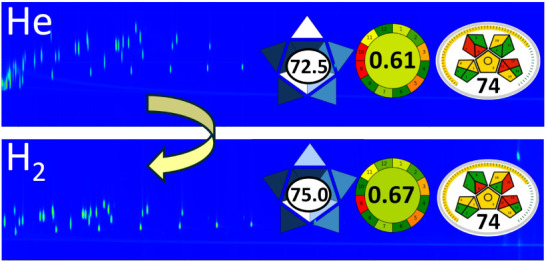

## Introduction

Carrier gas serves as the mobile phase that transports analytes through the column for separation in one-dimensional gas chromatography (1D GC) and comprehensive two-dimensional gas chromatography (GC×GC). Helium is traditionally used due to its inertness, but it is a finite resource obtained from natural gas with associated environmental impacts, including CO_2_ emissions and potential water contamination.^1,2^ Additional costs arise from extraction, transport, and handling of pressurized cylinders. As helium reserves decline and demand increases, prices have risen, limiting accessibility, particularly for routine or remote laboratory operations, and creating disparities across applications.^1,3^ Although experienced users can translate methods from helium to hydrogen using advanced knowledge and tools, there is a growing need for simplified workflows to support end users in this transition.

Hydrogen carrier gas offers distinct advantages for GC compared to helium. As described by the van Deemter equation, hydrogen enables comparable efficiency to helium at a faster optimal linear velocity due to its lower viscosity and higher diffusivity.^4,5^ This allows shorter analysis times without the loss of efficiency typically accompanied by increased helium flow rates, as observed by a flatter van Deemter curve for hydrogen than helium. For GC systems that use mass spectrometry (MS) detection, hydrogen is considered to be the most practical alternative to helium. Although nitrogen can be used in some specialized GC-MS applications, it is typically accompanied by a substantial decrease in S/N for conventional electron ionization.^6^ Hydrogen is increasingly recognized as a critical alternative to helium for GC and GC×GC, mitigating challenges associated with helium’s limited availability and escalating cost.^7^

The flammability risk associated with hydrogen can be minimized through generation in a laboratory rather than obtaining it in compressed cylinders. Cylinders of hydrogen are typically produced through the process of steam-methane reforming. Hydrogen generators typically use electrolysis, where water is split into oxygen gas and hydrogen gas using an electric current.^8,9^ This generation process is more sustainable compared to nonrenewable, fossil fuel-derived alternatives for hydrogen gas production.^8^ Gas transportation costs are eliminated with in-house generation since the gas is produced and subsequently consumed in real time. The flammability risk is lower with in-house generation due to the reduced storage in pressurized cylinders avoiding the accumulation of flammable gas.^9^ Additionally, hydrogen poses a higher risk of reactivity with analytes in the sample. While analyte conversion is not well documented in the literature with hydrogen carrier gas, chemical reactivity considerations dictate that there could be a possible risk of reduction of oxygen-containing groups or hydrogenation of double bonds in aromatic or vinyl groups. Especially for nontargeted analyses performed using GC×GC, monitoring analyte identification during the translation process can help characterize challenges for the annotation of components.

While tools exist for translating 1D GC methods to hydrogen carrier gas, no established approaches are available for GC×GC.^10–12^ One study compared key GC parameters for helium and hydrogen but did not provide translation guidance.^13^ Existing 1D translation approaches do not account for the additional programmable parameters of GC×GC separations. The modulator and fast secondary separation introduce zones with parameters not addressed in 1D GC studies converting helium to hydrogen carrier gas, leaving a gap in understanding how to optimize hydrogen carrier gas for GC×GC-MS separations.

The purpose of this work was to develop and evaluate an approach for converting cryogenically modulated GC×GC-MS systems from helium to hydrogen carrier gas. Chemical standards and a 1D translation tool were used to investigate parameters governing second dimension separations and chromatographic performance. The approach was subsequently tested on authentic fingermark samples as a forensic nontargeted profiling application.

## Materials and Methods

### Method Translation

This study evaluated three chemical standards that represent analytes commonly encountered in volatile organic compound (VOC) analysis. A 52-Component Indoor Air standard (95:5 MeOH:H_2_O, 100 ppm, Sigma-Aldrich, Saint Louis, MO, USA), a Grob standard (“ready-to-use” in hexane/dichloromethane, Sigma-Aldrich, Saint Louis, MO, USA), and a C_7_–C_30_ saturated linear alkanes mix (10 ppm in hexane, Sigma-Aldrich, Saint Louis, MO, USA) were used.

Compound reactivity, resolution values for peak pairs, and other component metrics (e.g., peak area, S/N, tailing factor) were compared. The Pro EZGC Method Translator (Restek Corporation, Bellefonte, PA, USA) was implemented to provide three options: translate, efficiency, and speed. Standards were analyzed using helium and all three hydrogen method options. The helium method and its three respective hydrogen methods are shown below for the indoor air standard (Table 1) and in the Supporting Information for Grob and linear alkane standards (Tables S1–S2). Resulting parameters from the tool were entered as whole values into ChromaTOF software 5.58. The first-dimension peak capacity, second-dimension peak capacity, and net peak capacity of the method were calculated using the Simply GC×GC software (LECO Corporation).

**Table 1:** Method Parameters Used for the Analysis of a 52-Component Indoor Air Standard

Parameter	Helium	H_2_-Translate	H_2_-Speed	H_2_-Efficiency
Column Flow (mL/min)	1.00	1.25	2.50	1.75
Average Velocity (cm/s)	36.12	60.24	85.19	71.27
Hold Up Time (min)	1.38	0.83	0.59	0.70
Inlet Pressure (psi)	7.38	1.85	8.70	4.88
Vacuum Pressure (psi)	0	0	0	0
Start Temperature (°C)	40	40	40	40
Hold Time Start (min)	3.0	1.8	1.25	1.5
Ramp (°C/min)	5.0	8.3	11.8	9.9
Final Temperature (°C)	250	250	250	250
Hold Time End (min)	1.0	0.6	0.4	0.5
Run Time (min)	46.00	27.70	19.45	23.20
Speed	1.00	1.66×	2.37×	1.98×
1D Peak Capacity	274	274	244	265
2D Peak Capacity	16	15	14	14
Net Peak Capacity	2728	2519	2166	2430

Analysis was performed on a Pegasus BT 4D GC×GC with time-of-flight MS (TOFMS) and dual-stage quad-jet cryogenic modulation (LECO Corporation, Saint Joseph, MI) using liquid injection. The syringe was rinsed with solvent and sample twice, and 1 μL of sample was injected, followed by five rinses. The inlet split ratio was 20:1, and the inlet temperature was 250 °C. Helium was of ultra high purity at 99.999% (Airgas, Radnor, PA, USA). Hydrogen was supplied at the specified flow rate and was generated by an NM Plus 600 Hydrogen Generator with a purity of 99.9996% (VICI DBS, Houston, TX) connected to the GC with stainless steel tubing, which is highly recommended for employing hydrogen carrier gas to reduce flammability risk.

The column set was identical for all methods. The primary column was a 30 m × 0.25 mm ID × 0.25 μm d_f_ Rxi-5 ms capillary column, and the secondary column was a 0.90 m × 0.25 mm ID × 0.25 μm d_f_ Rxi-17Sil MS capillary column (Restek, Bellefonte, PA). The primary oven temperature program was input according to Table 1, Table S1, and Table S2. GC×GC parameters included a 2 s modulation period (0.6 s hot pulse time/0.4 s cool time between stages), +5 °C secondary oven offset temperature, and +5 °C modulator offset temperature. The MS was operated with a 120 s acquisition delay for helium and a 180 s acquisition delay for hydrogen. The acquisition rate was 200 spectra/s, the mass range was 35–450 *m/z,* and the extraction frequency was 32 kHz. The transfer line and ion source temperatures were 270 °C. Electron ionization energy was 70.0 eV, and the emission current was 1.0 mA.

### Fingermark Residue Application

Fingermark residue samples were tested with helium and hydrogen as examples of a nontargeted forensic application. Seven standards were combined for the translation of a GC×GC-TOFMS method established in the literature,^14^ including myristic acid, octisalate, palmitoleic acid, palmitic acid, oleic acid, stearic acid, and methyl nonadecanoate (Millipore Sigma, Burlington, MA). Authentic fingermark samples were collected according to a protocol approved by William & Mary IRB (PHSC-2024-03-05-16929-kaperrault).^14^ The Pro EZGC Method Translator (Restek) was used to convert the established method to hydrogen carrier gas under the same workflow (Table 2).^12,14^

**Table 2:** Method Parameters Used for the Analysis of Fingermark Samples

Parameter	Helium	H_2_-Translate	H_2_-Speed	H_2_-Efficiency
Column Flow (mL/min)	1.00	1.25	2.50	1.75
Average Velocity (cm/s)	36.80	61.36	86.78	72.60
Hold Up Time (min)	1.36	0.81	0.58	0.69
Inlet Pressure (psi)	9.74	3.62	11.21	6.98
Vacuum Pressure (psi)	0	0	0	0
Start Temperature (°C)	80	80	80	80
Hold Time Start (min)	1.0	0.6	0.4	0.5
Ramp (°C/min)	5.0	8.3	11.8	9.8
Final Temperature (°C)	300	300	300	300
Hold Time End (min)	10.00	6.00	4.25	5.10
Run Time (min)	55.00	33.11	23.29	28.05
Speed	1.00	1.66×	2.36×	1.96×

Dichloromethane was used as the rinse solvent. A 5 s modulation period (1.5 s hot pulse time/1.0 s cool time between stages), +10 °C secondary oven offset temperature, and +15 °C modulator offset were used. The acquisition delay was 180 s and a 200 spectra/s acquisition rate was used. The mass range was set to 35–500 *m*/*z* with an extraction frequency of 32 kHz. The ionization energy was 70.0 eV with a 1.0 mA emission current.

Samples were analyzed by using ChromaTOF version 5.58 (LECO Corporation). Spectra were searched in the NIST MS Library Version 3.0, 2023. Samples were processed with a minimum S/N of 400 and a stick count of 3. The minimum spectral similarity was 700, and the relative abundance threshold was 10. Putative identifications were compared between methods to check the reactivity of components.

### Greenness Evaluation

Analytical greenness metrics were evaluated using automated sample preparation and associated analytical steps, incorporating the power consumption of the hydrogen generator. The Blue Applicability Grade Index (BAGI) (https://bagi-index.anvil.app/),^15^ the Modified Green Analytical Procedure Index (MoGAPI) (https://fotouhmansour.github.io/MoGAPI/),^16^ and the AGREE metric (https://agreeprep.anvil.app/) were used.^17^

## Results and Discussion

### Hydrogen Method Translation—Standards

The ProEZGC Method Translator allows the input of an established 1D GC helium method to solve for different goals with hydrogen: translate, efficiency, and speed (Figure S1).^12^ The translate option uses a combination of flow and temperature adjustments that match column efficiency between the original and new methods. The efficiency option is based on efficiency-optimized flow, which generates optimal peak capacity.^4,18^ Finally, the speed option uses speed-optimized flow with an optimal heating rate (OHR) and will yield the highest peak capacity possible per unit of time, often resulting in a shorter run with less-than-ideal resolution.

The hydrogen translate, efficiency, and speed methods reduced run time by ∼1.66×, ∼1.96×, and ∼2.36×, respectively, with expected concessions to the first dimension and net peak capacity (Table 1). Chromatographic performance was evaluated using the resolution of key peak pairs, peak tailing, peak area, and signal-to-noise (S/N) as calculated in the ChromaTOF software. Peak tailing in the software is calculated as the right width divided by the left width at 10% height. The translate method exhibited the best use of the chromatographic space for all standards. An example is shown for the indoor air standard (Figure 1). The speed option displayed the least optimal peak shapes visually, as expected, which was verified by tailing values (Table 3). The efficiency method improved peak shapes from the speed option, and the translate option displayed the best peak shapes (Table 3). Although requiring a few extra minutes for the translate option, it resulted in the lowest peak tailing for analytes while still reducing run time by 60%. There was a small increase in peak tailing for select analytes between the helium and hydrogen methods.

**Figure 1:**
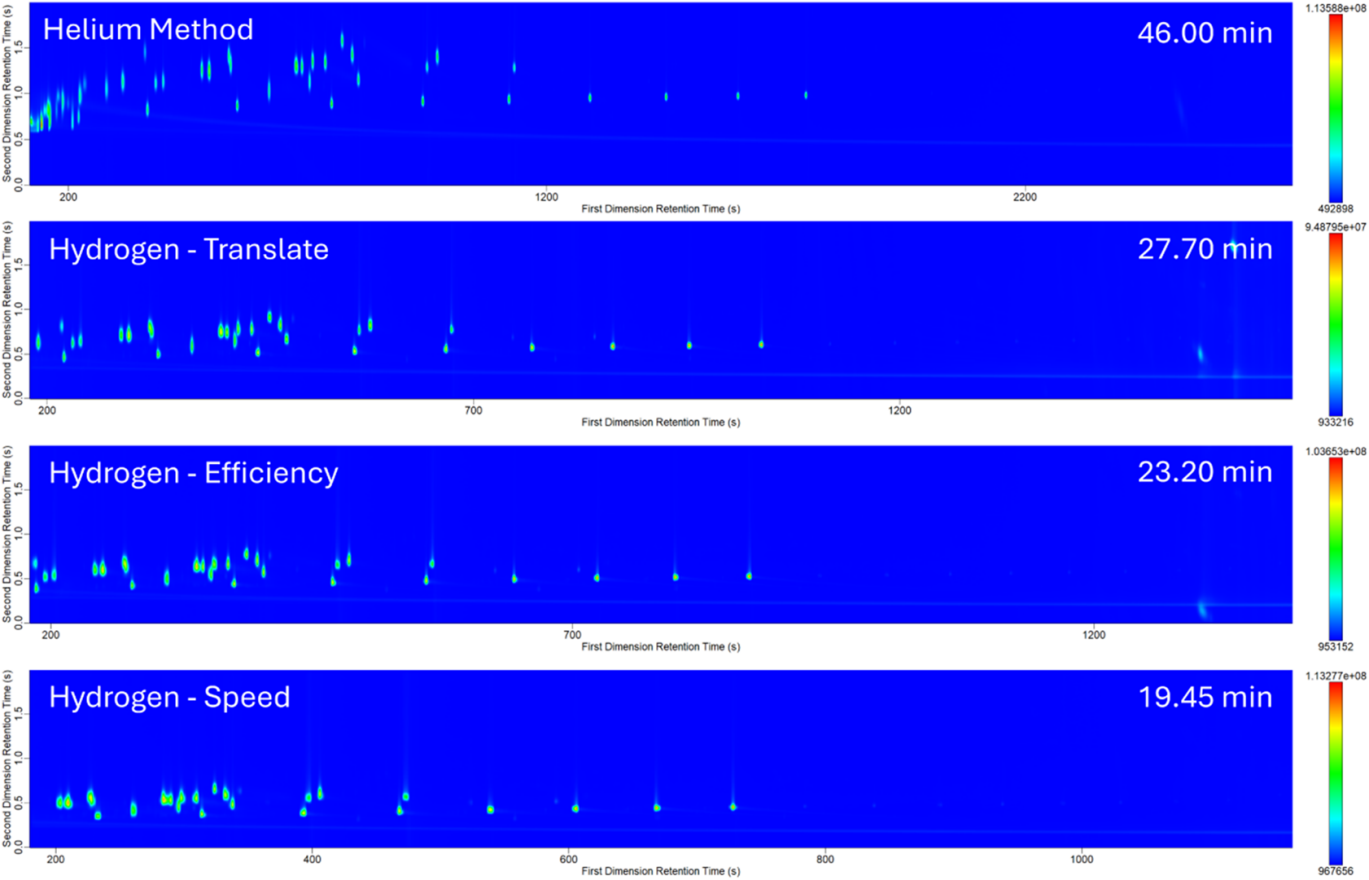
52-Component Indoor Air standard total ion current contour plots run with the output of the Restek Method Translator (translate, efficiency, and speed methods).

**Table 3:** Tailing Factor for Representative Analytes from the Indoor Air Standard Using Different Hydrogen Method Conversion Options

Analyte	Helium	H_2_-Translate	H_2_-Efficiency	H_2_-Speed
β-Pinene	1.21	1.38	1.45	1.54
Decanal	1.23	2.11	2.38	2.12
Hexadecane	1.08	1.65	1.71	1.89
Limonene	1.23	1.37	1.60	1.69
*p*-Xylene	1.16	1.25	1.65	1.42
Styrene	1.19	1.32	1.36	1.35
Tridecane	1.06	1.09	1.83	1.43
Undecane	1.29	1.53	1.84	1.90

Peak area and S/N were evaluated for compounds across multiple classes (Tables S3–S4). The translate option provided the most favorable area and S/N, though trends were not consistent across analytes. For example, β-pinene showed higher S/N with the translate method, while styrene exhibited comparable S/N across all methods. Peak resolution was also assessed for key pairs using first-dimension, second-dimension, and combined 2D metrics. Examples show comparable resolution for the translate method option (Figure 2). Differences between methods were minimal for peaks already well resolved in either dimension (Figure S2).

**Figure 2:**
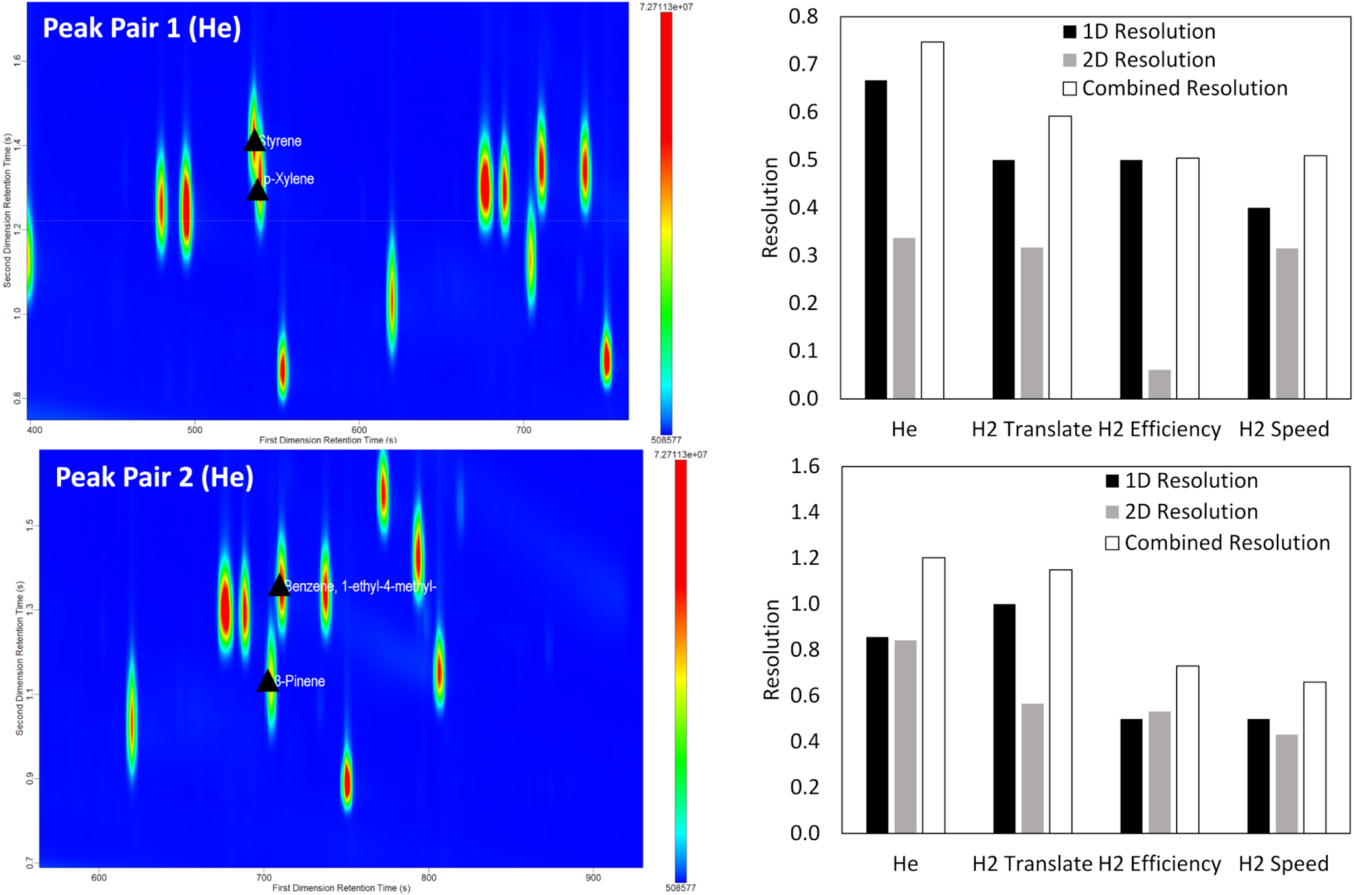
Peak resolution total ion current (TIC) contour plot results in the first dimension (1D), second dimension (2D), and combined dimensions. Pair 1: styrene and *p*-xylene; Pair 2: 1-ethyl-4-methylbenzene and β-pinene.

The first investigation of hydrogen for GC×GC-TOFMS in the literature noted a 25% reduction in primary dimension time based on theoretical Golay curves and optimal plate height.^13^ The 60% time reduction in this study demonstrated adequate resolution for the separation of analytes while increasing energy efficiency with shorter analysis. Faster analyses are beneficial especially to laboratories such as forensic or food control laboratories which must routinely analyze batches of samples to meet time-sensitive deadlines.

The higher hydrogen flow caused early-eluting analytes to elute before MS acquisition began (Figure 1, Figures S3 and S4). When translating methods, a shorter acquisition delay should be used. The column configuration was kept constant to enable direct comparison, though its impact on translation warrants further study. Maintaining the same configuration benefits laboratories that switch between carrier gases or assess the analyte reactivity with hydrogen.

The modulation period was initially kept constant for translation, then selected based on complete second dimension elution (“proposed modulation period”). As expected, hydrogen produced faster second-dimension separations. Second dimension retention times decreased in an approximately 2:1 ratio (Figure 3, Table 4), indicating that the modulation period can be reduced by ∼50% when switching to hydrogen, assuming no column changes. This trend was confirmed for all standards (Figure 1, Figures S3–S4) and represented the first reported ratio to guide modulation period adjustment during GC×GC carrier gas translation. While this work solely deals with cryogenic modulation, flow-modulated systems are more complex and should be investigated further to identify if this impact is similar. Additionally, the number of modulations per peak was preserved when moving from helium to hydrogen carrier gas (Figure S5).

**Figure 3:**
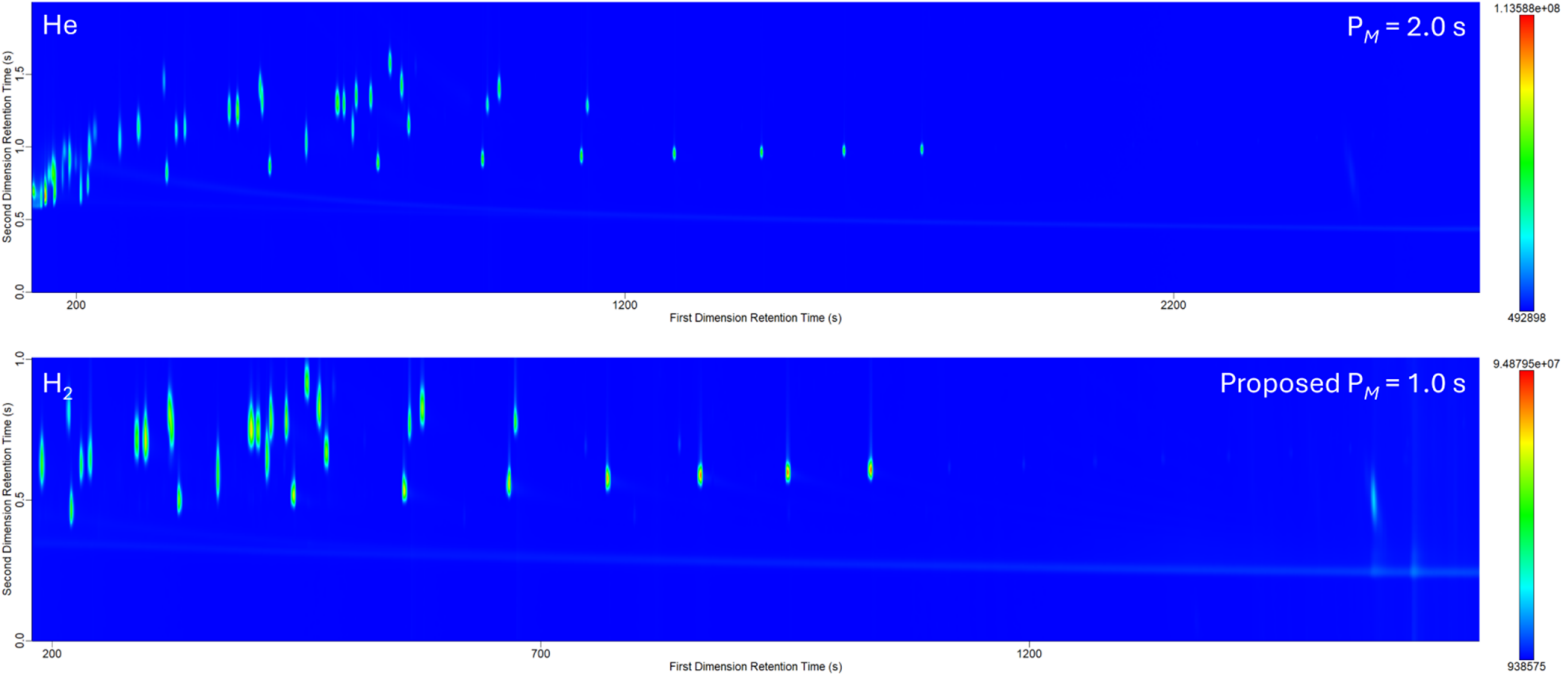
52-Component Indoor Air standard total ion current (TIC) contour plots using both carrier gases showing the proposed modulation period.

**Table 4:** Modulation Periods (P_*M*_) for Helium and Proposed Modulation Period for Hydrogen with Percent Reduction

Sample	P_*M*_ (He)	Proposed P_*M*_ (H_2_)	% Reduction
C_7_–C_30_ Alkanes	2.0 s	1.0 s	50
Grob Standard	3.0 s	1.5 s	50
52-Component Indoor Air Standard	2.0 s	1.0 s	50
Fingermark Residue	5.0 s	2.5 s	50

### Greenness Evaluation

Three metrics for evaluating analytical greenness were used to compare carrier gases used in this study (Figure 4).^19^ The Blue Applicability Grade Index (BAGI) visually identifies points of methodology by ranking parameters of practicality and applicability in a blue color-scale asteroid pictogram ranging from 0 to 100.^15^ The applicability of a method is inherently sustainable; a procedure that is more applicable to a wide variety of analytes or uses fewer resources is more sustainable. The Modified Green Analytical Procedure Index (MoGAPI) was used to evaluate aspects of greenness throughout multiple stages of analysis including sample preparation, solvent usage, and instrumentation.^16^ Finally, the AGREE metric scores an analytical procedure from 0 to 1 based on 12 criteria corresponding to the principles of green chemistry.^17^ The hydrogen translate option was used in metric calculations.

**Figure 4:**
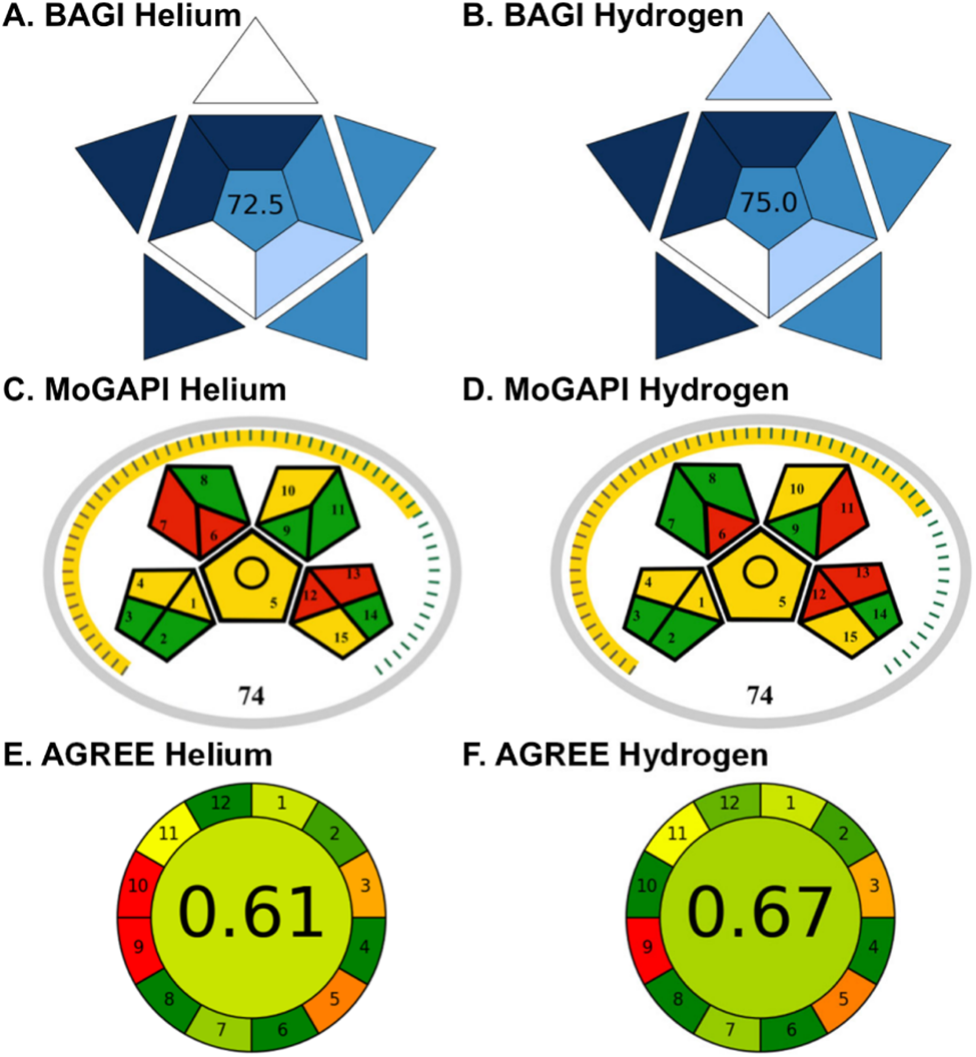
Comparison of green analytical metrics for GC×GC-TOFMS methods using helium and hydrogen carrier gases: (A) BAGI helium, (B) BAGI hydrogen, (C) MoGAPI helium, (D) MoGAPI hydrogen, (E) AGREE helium, and (F) AGREE hydrogen.

The hydrogen method achieved a higher BAGI score (Figure 4B) than helium (Figure 4A), driven by increased sample throughput. The faster optimal linear velocity enabled a 50% reduction in run time (Figure 1), doubling throughput and reducing energy consumption by 2636 W per sample (Figure 4A–B). Both gases produced identical MoGAPI scores, despite differences in Criteria 7 and 11 (Figure 4C–D). Criterion 7 (solvent origin) favored hydrogen, which was generated in-house via water electrolysis, and received a green score. In contrast, Criterion 11 (safety) penalized hydrogen due to its flammability, while helium received a favorable rating. The hydrogen method achieved a higher AGREE score than helium (Figure 4E–F), driven by Criteria 10 and 12. Criterion 10 (renewability) favored hydrogen due to in-house generation, whereas helium scored lower. Criterion 12 (safety) penalized hydrogen for its flammability. Although Criterion 9 (energy consumption) did not distinguish between methods since both exceeded >500 W,^19^ the reduced run time with hydrogen has a practical impact. For example, hydrogen enables ∼52 samples per day compared to ∼31 samples for helium, highlighting meaningful gains in throughput and energy efficiency for routine analyses.

The analysis time reduction using hydrogen carrier gas has been noted as positive for the sustainability and efficiency of routine laboratories analyzing steroids, explosives, and pesticides.^10,11,20^ A developed method for the analysis of dioxin-like polychlorinated biphenyls similarly noted lower energy consumption per sample and increased sample throughput.^20^ This is highlighted across green analytical metrics despite small changes in overall scoring. Switching to hydrogen as a carrier gas can nearly double the sample throughput, reducing analysis time and operational costs; however, sustainability comparisons with helium are nuanced. A broader perspective should include efficiency, cost, and practical considerations, such as reduced gas deliveries and cylinder handling.

### Fingermark Residue Application

Seven analytes (fatty acids and esters) were used to form an artificial fingermark residue and assess peak shape and reproducibility. These compounds were selected due to their known variability in the peak shape. Although acids often exhibit tailing without derivatization, derivatization was not used to reflect nontargeted workflows.

The analytes (Figure 5A–C) showed mixed trends for tailing factor, exhibiting both improved and worsened tailing across methods (Figure 5). Increased peak tailing was found in the analysis of adulterated drugs in traditional Chinese medicines using hydrogen carrier gas compared to helium.^9^ Conversely, hydrogen produced the best peak shape among the three tested carrier gases in another study on permanent gas detection in human blood.^21^ The results of this study demonstrated comparable tailing factors relative to helium depending on analyte chemistry.

**Figure 5:**
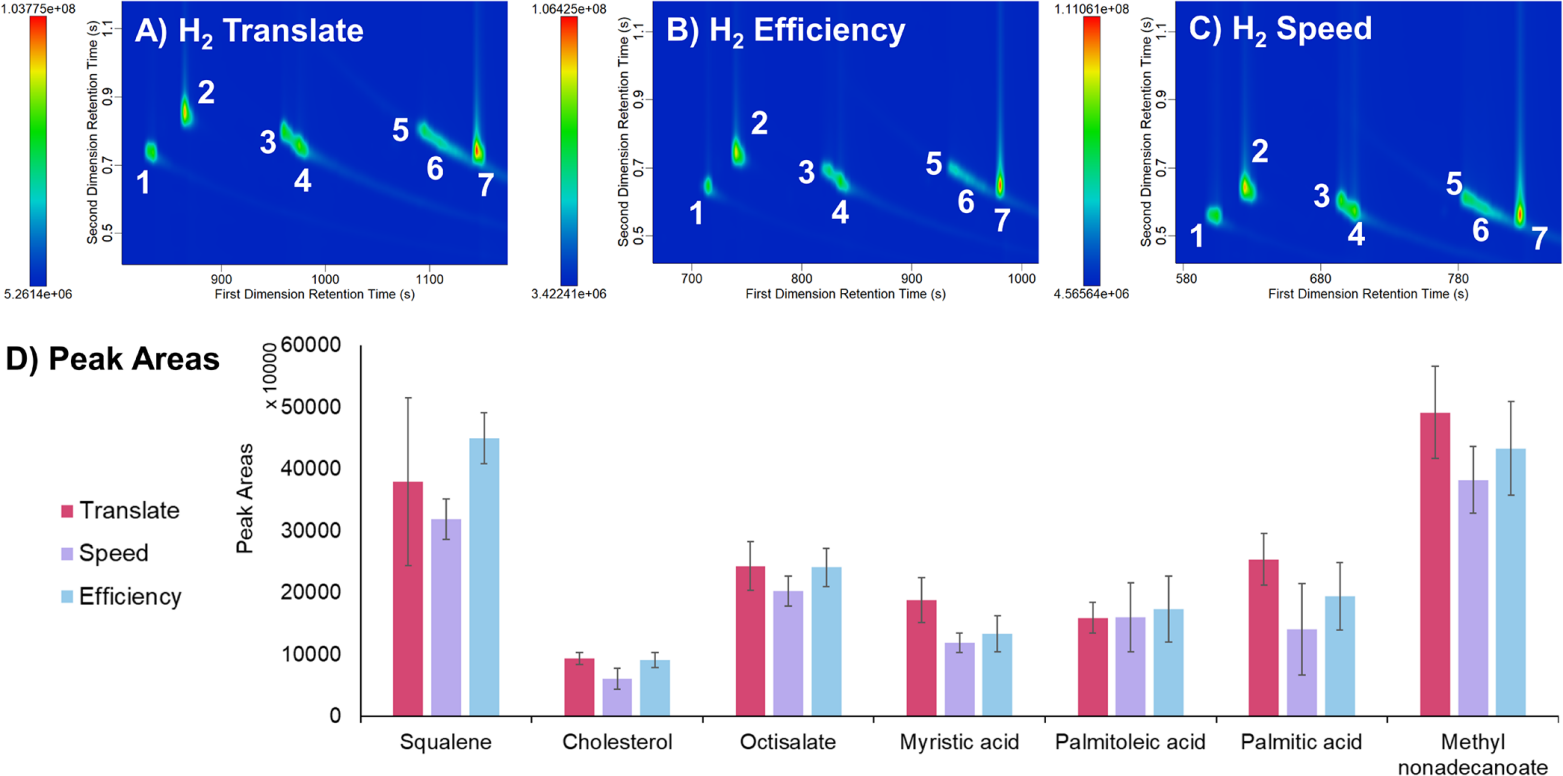
Analytical ion current (AIC) contour plots of fingermark standards run using hydrogen carrier gas: (A) H_2_ translate, (B) H_2_ efficiency, (C) H_2_ speed. D) Peak areas normalized to seven analytes (*n* = 9). 1) myristic acid, 2) octisalate, 3) palmitoleic acid, 4) palmitic acid, 5) oleic acid, 6) stearic acid, and 7) methyl nonadecanoate.

A key difference between the three hydrogen methods was the flow rate of the carrier gas. The helium flow rate of 1.00 mL/min was translated to 1.25 mL/min of gas flow for hydrogen. The speed method resulted in a flow of 2.50 mL/min, while the efficiency method resulted in a flow rate of 1.75 mL/min. The translate, efficiency, and speed method options had the same improved respective chromatographic run times as the standards reported above (Table 2). Studies using GC-MS that investigated drug samples found that switching to hydrogen carrier gas from helium decreased run time by more than half, thereby benefiting throughput.^2,9^ Decreased first-dimension retention times (faster elution) were expected with higher flow rates and provided similar results as above.

Significant differences were observed across methods for squalene, cholesterol, octisalate, myristic acid, palmitic acid, and methyl nonadecanoate using a one-way ANOVA test (*p* < 0.05) (Figure 5D). The translate method generally indicated a stronger response for many analytes and improved reproducibility for some analytes (Figure 5D). These trends align with previous 1D GC-MS studies reporting comparable reproducibility between hydrogen and helium for pesticide analysis.^20^ The translate method was selected for the analysis of authentic fingermark samples.

Fingermark residue from five volunteers was analyzed using all three hydrogen methods to confirm method selection. The translate method showed the least peak tailing (Figure 6A–C) and improved suitability for quantitative analysis. Several analytes exhibited significant first-dimension tailing with the efficiency method (Figure 6B). The proposed modulation period avoided wraparound based on second-dimension retention times (Figure S6). Wraparound peaks that do not reflect true first or second dimension retention times should be avoided in GC×GC analysis to avoid retention index challenges and second-dimension broadening.^22^ The modulation period was reduced by 50%, similar to the standards tested above (Table 3, Figure S6). The same metrics used for standards were also applied to authentic fingermark samples, yielding consistent results.

**Figure 6:**
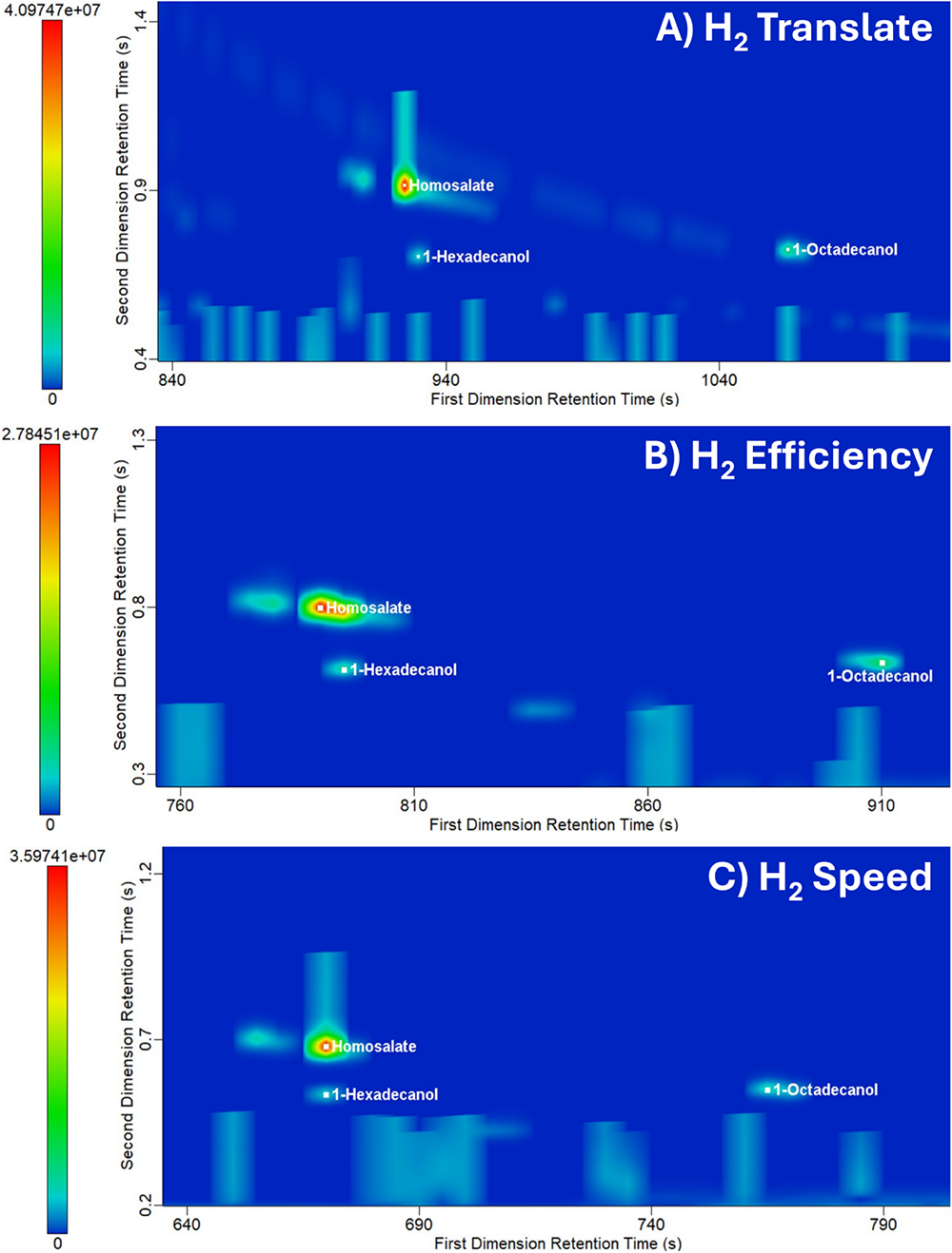
Analytical ion current (AIC) contour plots of fingermark residue analytes using A) hydrogen translate, B) hydrogen efficiency, and C) hydrogen speed methods.

One concern is whether hydrogen interferes with MS fragmentation patterns through reactions of analytes in the system. For the standards and fingermark residue samples, all detected analytes were identified using both carrier gases with retention times reported in Supporting Information (Tables S5, S6, and S7). Previous GC-MS studies have similarly demonstrated successful use of hydrogen carrier gas across a range of applications including illicit drugs^11^ Studies of explosives and dioxin-like polychlorinated biphenyls found slight differences in electron ionization (EI) ion ratios using hydrogen carrier gas but similar overall fragmentation patterns, and one author suggested MS databases specifically for hydrogen.^11,23^ Other studies found slight differences in EI fragmentation between mass spectra collected from GC-MS analysis using hydrogen carrier gas compared to helium for certain analytes, but these minor differences did not hinder analyte identification using libraries.^9,10,13^ Other researchers have demonstrated comparable results between hydrogen and helium carrier gas for food control laboratories investigating pesticide contamination and VOC composition of pepper species.^13,20^ Herein, no challenges were observed in identifying any compounds with hydrogen carrier gas. Generated hydrogen is typically higher purity (99.9996%) than ultra-high-purity helium (99.999%) which may reduce impurities in the analysis. The structured chromatographic organization provided by GC×GC may offer an inherent advantage when adopting alternative carrier gases, as retention behavior across two dimensions provides complementary chemical information that could help support analyte identification should carrier-gas-related spectral differences occur in the ion source. Researchers should continue to monitor analyte chemistry when switching to hydrogen carrier gas, since this study does not cover all possible analyte classes. Future work could also assess column or source reactivity through deactivation strategies or by assessing source cleanliness. A workflow outlining the steps to assess the carrier gas change is shown (Figure 7). Using 1D GC translation tools, users can convert an optimized method to hydrogen carrier gas by following these steps.

**Figure 7:**
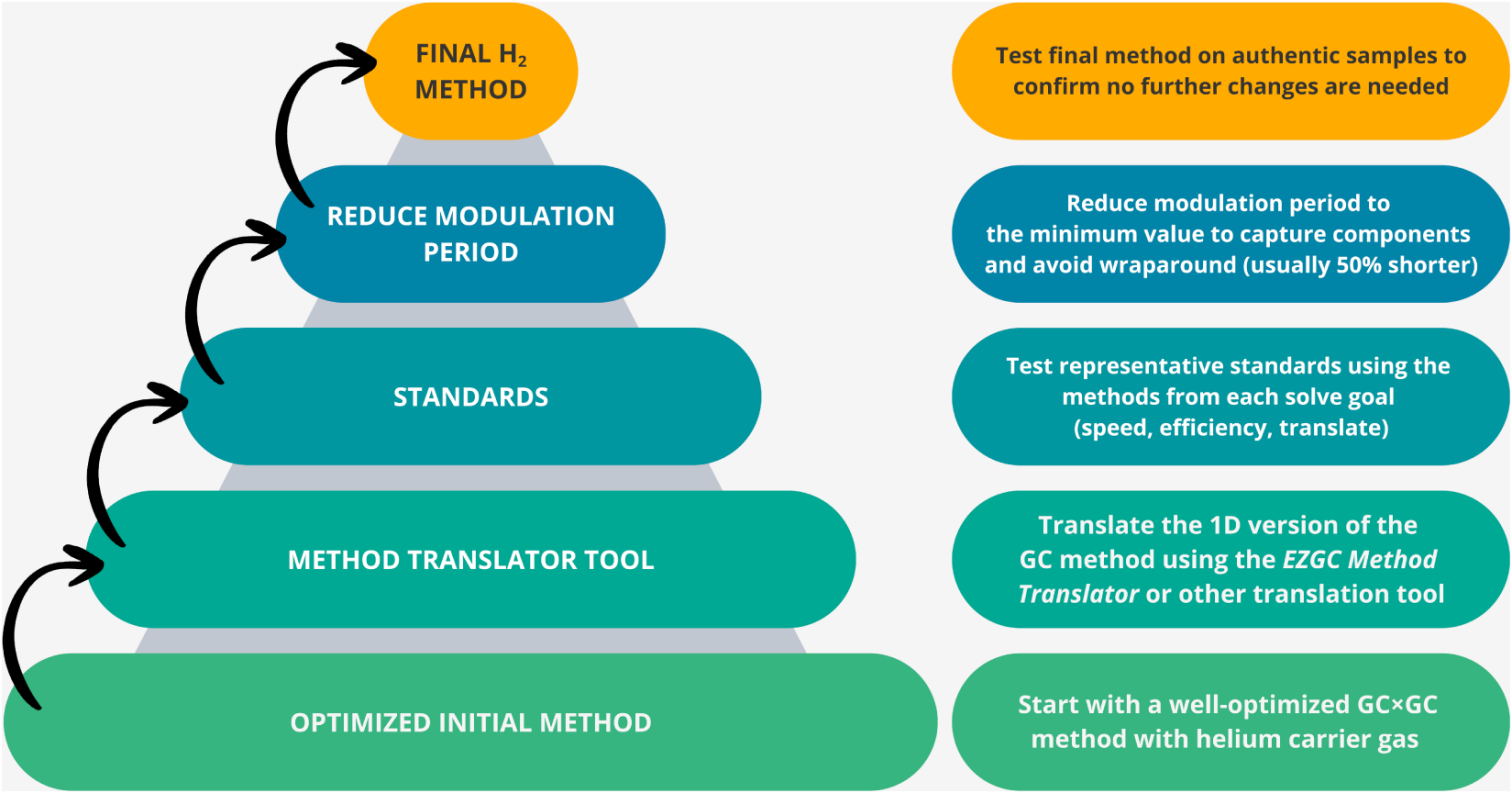
Conversion of a GC×GC method from helium to hydrogen carrier gas.

## Conclusion

Helium has long been the standard carrier gas for GC-MS due to its inertness but has become increasingly expensive and challenging to acquire. Hydrogen is an attractive alternative carrier gas because of its lower viscosity and higher diffusivity relative to helium, allowing comparable chromatographic efficiency to be maintained over a wider range of linear velocities. Consequently, hydrogen can support faster separations with reduced loss of efficiency than helium at elevated flow rates. This characteristic has made hydrogen an increasingly viable carrier gas for GC and GC×GC applications. This study used analytical standards to develop a translation workflow from helium to three hydrogen methods using GC×GC-TOFMS with a cryogenic modulator and existing 1D GC translation tools. The translate option displayed the best peak shape and reproducibility over the speed or efficiency options. The run time was reduced by 60% using hydrogen compared to helium, and the modulation period could be reduced by 50%. This translation was performed with fingermark standards and authentic fingermark samples to verify the workflow. This study is the first to propose a retention time reduction ratio for translating a GC×GC-TOFMS helium method to hydrogen in cryogenically modulated systems.

Faster analyses using hydrogen carrier gas align with the green chemistry principle of energy efficiency. Shorter run times reduce electricity use per sample and increase overall throughput. The hydrogen method was comparable to its helium counterpart while reducing per-run energy consumption and nearly doubling hourly sample throughput. This provides clear advantages for routine, high-throughput laboratories. Hydrogen also offers potential sustainability benefits through on-site generation via electrolysis, in contrast to the nonrenewable sourcing of helium. The overall sustainability impact reflects a balance of factors including energy use, gas sourcing, safety considerations, and operational efficiencies. Hydrogen presents a compelling option when evaluated through this broader lens. The findings from this study were found to be replicated in real fingermark samples, making this applicable to other authentic samples analyzed using GC×GC-TOFMS.

## Supplementary Material



## Data Availability

The data are available from the corresponding author upon reasonable request.

## Notes

GC parameters used for standards, retention time comparison for analytes, translation tool outputs, and sample TIC data (PDF)
