# Supplementary material for: Hydrogen Carrier Gas Method Translation in Comprehensive Two-Dimensional Gas Chromatography for Sustainable Nontargeted Analysis
Source: Anal Chem. 2026 Jul 17;98(30):22025–33. doi: 10.1021/acs.analchem.6c03350 (PMC13449218; doi:10.1021/acs.analchem.6c03350)
Supplement: Supplementary file 1 [file ac6c03350_si_001.pdf]

Supporting Information

Hydrogen carrier gas method translation in comprehensive two-dimensional gas chromatography for sustainable nontargeted analysis

Kira M. Fisher [1], Emma L. Macturk[1], Katelynn A. Perrault Uptmor[1*]
[1]Nontargeted Separations Laboratory, Chemistry Department, William & Mary

*Corresponding Author
Katelynn A. Perrault Uptmor
Nontargeted Separations Laboratory, Chemistry Department
William & Mary
Integrated Science Center 1053
540 Landrum Drive
Williamsburg, VA 23188
USA
kaperrault@wm.edu
757-221-4793

SI Table of Contents

Table S1. GC parameters from a reference helium GC×GC method and three generated hydrogen carrier gas methods from the translator tool for analysis of Grob standard S3
Table S2. GC parameters from a reference helium GC×GC method and three generated hydrogen carrier gas methods from the translator tool for analysis of C7-C30 alkane standard S3
Table S3. Peak S/N for representative analytes from the indoor air standard using different hydrogen conversion options S4
Table S4. Peak Area for representative analytes from the indoor air standard using different hydrogen conversion options S4
Table S5. Retention time comparison for the alkane standard run with helium and hydrogen. First dimension retention time (1tR) and second dimension retention time (2tR) are shown for both helium (He) and hydrogen (H2) in seconds S5
Table S6. Retention time comparison for the alkane standard run with helium and hydrogen carrier gas. First dimension retention time (1tR) and second dimension retention time (2tR) are shown for both helium (He) and hydrogen (H2) in seconds S6-S7
Table S7. Retention time comparison for the Grob standard run with helium and hydrogen. First dimension retention time (1tR) and second dimension retention time (2tR) are shown for both helium (He) and hydrogen (H2) in seconds. Values are reported to the same number of significant digits reported in the software S7
Figure S1. Restek EZGC Method Translator outputs for the efficiency, speed, and translate methods for a 52-component Indoor Air standard hydrogen method S8
Figure S2. Peak resolution results in first dimension (1D), second dimension (2D) and combined when comparing two examples of peak pairs. Peak pair 3 shows styrene and nonane while peak pair 4 shows ethylbenzene and p-xylene S8
Figure S3. Grob standard contour plots ran on helium (top) and hydrogen (bottom) showing modulation period used and proposed modulation period for a hydrogen translation. The proposed modulation period is shown on the y-axis on the chromatogram S9
Figure S4. Alkane standard contour plots ran on helium (top) and hydrogen (bottom) showing proposed modulation period for a hydrogen translation. The proposed modulation period is shown on the y-axis on the chromatogram S9
Figure S5. Modulated subpeaks for α-pinene and toluene shown as a 1D chromatogram and corresponding contour plots for each peak S10
Figure S6. Fingerprint sample contour plots ran on helium (top) and hydrogen (bottom) showing proposed modulation period for a hydrogen translation. The proposed modulation period is shown on the y-axis on the chromatogram. The chromatograms are zoomed in to show the latter half of the chromatogram because analytes are much heavier than the analytes in the other standards S11

Table S1. GC parameters from a reference helium GC×GC method and three generated hydrogen carrier gas methods from the translator tool for analysis of Grob standard.

Parameter	Helium	H_2 Translate	H_2 Speed	H_2 Efficiency
**Column Flow (mL/min)**	1.00	1.00	2.50	1.75
**Average Velocity (cm/s)**	36.12	36.12	85.19	71.27
**Hold Up Time (min)**	1.38	1.38	0.59	0.70
**Inlet Pressure (psi)**	7.38	7.38	8.70	4.88
**Vacuum Pressure (psi)**	0	0	0	0
**Start Temperature (°C)**	40	40	40	40
**Hold Time Start (min)**	2.0	1.2	0.85	1.0
**Ramp (°C/min)**	5	8.3	11.8	9.9
**Final Temperature (°C)**	250	250	250	250
**Hold Time End (min)**	1.0	0.6	0.4	0.5
**Run Time (min)**	45.00	27.10	19.05	22.71
**Speed**	1.00x	1.66x	2.36x	1.98x

Table S2. GC parameters from a reference helium GC×GC method and three generated hydrogen carrier gas methods from the translator tool for analysis of C_7-C_{30} alkane standard.

Parameter	Helium	H_2 Translate	H_2 Speed	H_2 Efficiency
**Column Flow (mL/min)**	1.00	1.25	2.50	1.75
**Average Velocity (cm/s)**	36.12	60.24	85.19	71.27
**Hold Up Time (min)**	1.38	0.83	0.59	0.70
**Inlet Pressure (psi)**	7.38	1.85	8.70	4.88
**Vacuum Pressure (psi)**	0	0	0	0
**Start Temperature (°C)**	40	40	40	40
**Hold Time Start (min)**	1.0	0.6	0.4	0.5
**Ramp (°C/min)**	5	8.3	11.8	9.9
**Final Temperature (°C)**	250	250	250	250
**Hold Time End (min)**	3	1.8	1.25	1.5
**Run Time (min)**	46	27.70	19.45	23.21
**Speed**	1.00x	1.66x	2.37x	1.98x

Table S3. Peak S/N for representative analytes from the indoor air standard using different hydrogen method conversion options.

Analyte	Helium	H_2 Translate	H_2 Efficiency	H_2 Speed
**β-Pinene**	31338	35301	14780	64656
**Undecane**	20984	32397	4801	32432
**Tridecane**	26893	10801	6236	23019
**Styrene**	24725	19872	20349	26130
**p-Xylene**	11668	19678	19424	28971
**Limonene**	41581	22329	22136	65971
**Hexadecane**	21246	8161	5850	20352
**Decanal**	4414	2904	2050	3794

Table S4. Peak Area for representative analytes from the indoor air standard using different hydrogen method conversion options.

Analyte	Helium	H_2 Translate	H_2 Efficiency	H_2 Speed
**β-Pinene**	544994043	480771198	506813115	430203710
**Undecane**	505661811	417716846	377571519	448257640
**Tridecane**	388003966	400538061	373915646	473063397
**Styrene**	791748333	570080366	577780055	566761886
**p-Xylene**	1042427211	750660409	1205737872	1218171168
**Limonene**	491445136	369777743	298190666	298318479
**Hexadecane**	270204061	374537773	389136058	369096258
**Decanal**	148027772	139911924	159301691	188156069

Table S5. Retention time comparison for the alkane standard run with helium and hydrogen. First dimension retention time (^1t_R) and second dimension retention time (^2t_R) are shown for both helium (He) and hydrogen (H_2) in seconds.

Analyte	He - ^1t_R	He - ^2t_R	H_2 - ^1t_R	H_2 - ^2t_R
**octane**	313.988	0.77	219.997	0.467
**nonane**	469.978	0.839	329.99	0.504
**decane**	647.966	0.88	447.983	0.521
**undecane**	829.955	0.901	559.976	0.545
**dodecane**	1005.94	0.932	667.969	0.563
**tridecane**	1171.93	0.955	767.962	0.58
**tetradecane**	1329.92	0.966	861.956	0.589
**pentadecane**	1479.91	0.974	951.951	0.6
**hexadecane**	1621.9	0.983	1037.95	0.607
**heptadecane**	1755.9	0.994	1115.94	0.62
**octadecane**	1883.89	1.005	1193.94	0.627
**nonadecane**	2005.88	1.017	1265.93	0.64
**eicosane**	2121.87	1.029	1335.93	0.652
**heneicosane**	2233.86	1.046	1401.92	0.659
**docosane**	2341.86	1.062	1465.92	0.67
**tricosane**	2443.85	1.085	1527.91	0.685
**tetracosane**	2541.84	1.109	1585.91	0.704
**pentacosane**	2639.84	1.245	1641.91	0.776
**hexacosane**	2715.83	1.137		
**heptacosane**	2755.83	1.446		

Table S6. Retention time comparison for the indoor air standard run with helium and hydrogen carrier gas. First dimension retention time ($^{1}t_R$) and second dimension retention time ($^{2}t_R$) are shown for both helium (He) and hydrogen (H_2) in seconds.

Analyte	He - $^{1}t_R$	He - $^{2}t_R$	H_2 - $^{1}t_R$	H_2 - $^{2}t_R$
**acetone**	122	0.666		
**hexane**	143.998	0.664		
**2,4-dimethylpentane**	137.999	0.64		
**2-butanone**	151.998	0.811		
**ethyl acetate**	157.998	0.813		
**chloroform**	157.998	0.854		
**iso-octane**	209.994	0.681		
**1,1,1-trichloroethane**	175.996	0.817		
**heptane**	221.993	0.74		
**carbon tetrachloride**	187.996	0.817		
**butanol**	199.995	0.89		
**1,2-dichloroethane**	179.996	0.972		
**benzene**	187.996	0.934		
**trichloroethylene**	223.993	0.944		
**1,2-dichloropropane**	223.993	1.038		
**bromodichloromethane**	233.993	1.105		
**4-methyl-2-pentanone**	279.99	1.052		
**octane**	365.984	0.832	219.997	0.465
**toluene**	313.988	1.133	189.999	0.632
**n-butyl acetate**	397.982	1.138	239.996	0.645
**tetrachloroethene**	381.983	1.114	229.997	0.626
**dibromochloromethane**	359.985	1.455	217.998	0.813
**nonane**	553.972	0.87	329.99	0.502
**ethylbenzene**	479.977	1.258	285.993	0.716
**m-xylene**	493.976	1.256	295.993	0.721
**p-xylene**	539.973	1.318	321.991	0.746
**styrene**	535.973	1.412	319.991	0.81
**(1S)-(-)alpha-pinene**	619.968	1.03	369.988	0.59
**decane**	749.96	0.894	447.983	0.517
**1,3,5-trimethylbenzene**	793.957	1.427	411.985	0.743
**(-)-beta-pinene**	703.963	1.13	419.985	0.651
**2-ethyltoluene**			403.986	0.752
**1,2,4-trimethylbenzene**	793.957	1.427	439.983	0.78
**(R)-(+)-limonene**	805.956	1.153	479.981	0.669
**undecane**	941.947	0.912	561.976	0.53
**1,2,3-trimethylbenzene**	793.957	1.427	473.981	0.827
**1,4-dichlorobenzene**	771.958	1.58	461.982	0.911
**nonanal**	949.947	1.29	565.975	0.77
**dodecane**	1121.94	0.938	667.969	0.56

Table S6 continued. Retention time comparison for the indoor air standard run with helium and hydrogen carrier gas. First dimension retention time (^1t_R) and second dimension retention time (^2t_R) are shown for both helium (He) and hydrogen (H_2) in seconds.

Analyte	He - ^1t_R	He - ^2t_R	H_2 - ^1t_R	H_2 - ^2t_R
**decanal**	1131.94	1.282	673.968	0.778
**tridecane**	1289.93	0.956	769.962	0.573
**tetradecane**	1449.91	0.963	861.956	0.588
**pentadecane**	1599.91	0.973	953.95	0.596
**hexadecane**	1741.9	0.982	1037.95	0.606

Table S7. Retention time comparison for the Grob standard run with helium and hydrogen. First dimension retention time (^1t_R) and second dimension retention time (^2t_R) are shown for both helium (He) and hydrogen (H_2) in seconds. Values are reported to the same number of significant digits reported in the software.

Analyte	He - ^1t_R	He - ^2t_R	H_2 - ^1t_R	H_2 - ^2t_R
**2,3 butanediol**	311.988	1.28	200.999	0.788
**decane**	698.963	0.917	446.983	0.535
**octanol**	830.955	1.253	527.978	0.751
**2,6-dimethylphenol**	896.95	1.755	566.975	1.057
**hexanoic acid, 2-ethyl**	941.947	1.239	584.974	0.742
**2,6-dimethylaniline**	1007.94	1.99	632.971	1.202
**dodecane**	1064.94	0.941	668.969	0.57
**methyl decanoate**	1271.93	1.238	791.961	0.765
**dicyclohexylamine**	1421.92	1.41	884.955	0.849
**methyl undecanoate**	1430.92	1.227	887.955	0.758
**methyl dodecanoate**	1577.91	1.225	974.949	0.76

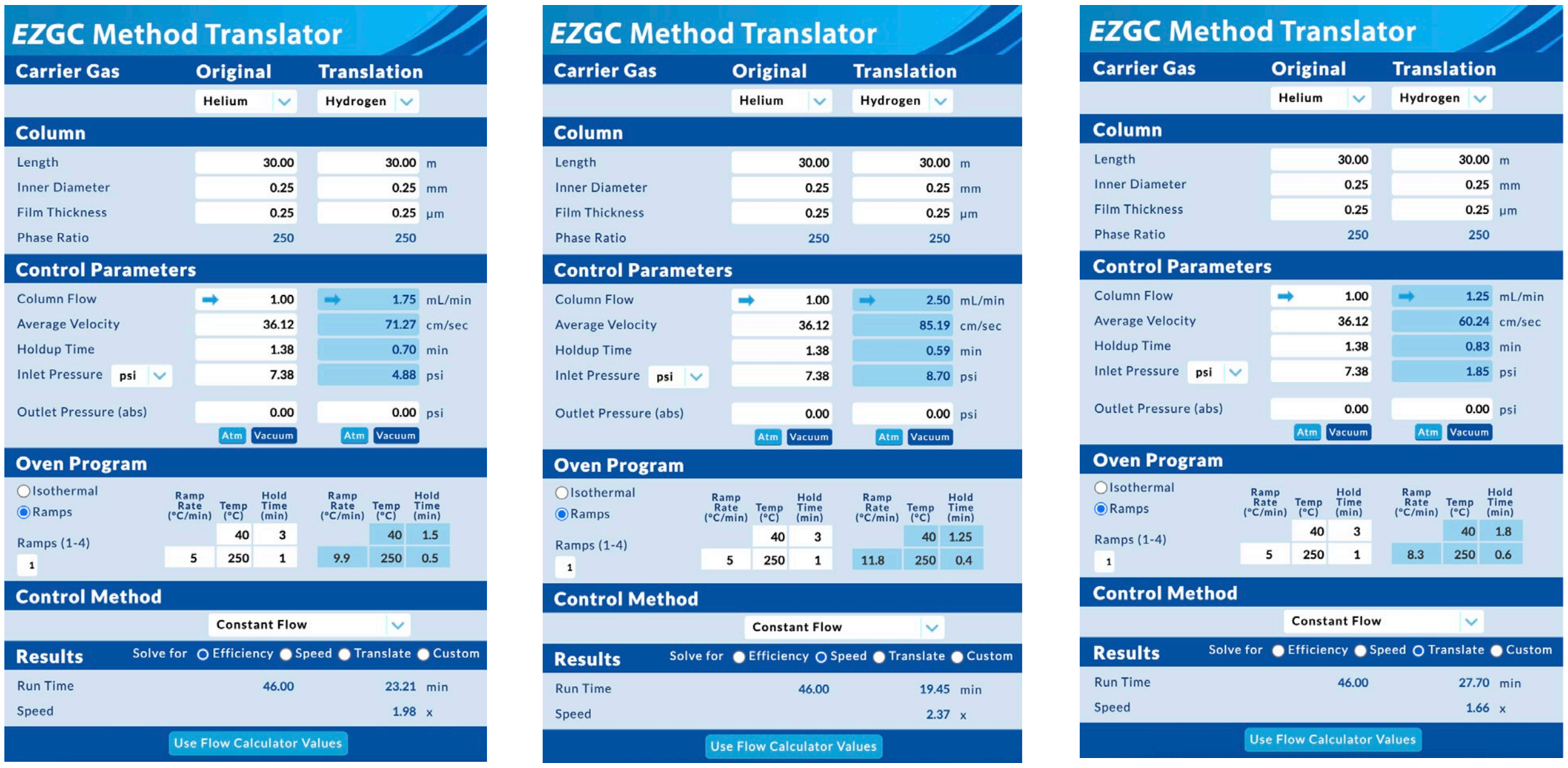

Efficiency **Speed** **Translate**

Figure S1. Restek EZGC Method Translator outputs for the efficiency, speed, and translate methods for a 52-component Indoor Air standard hydrogen method.

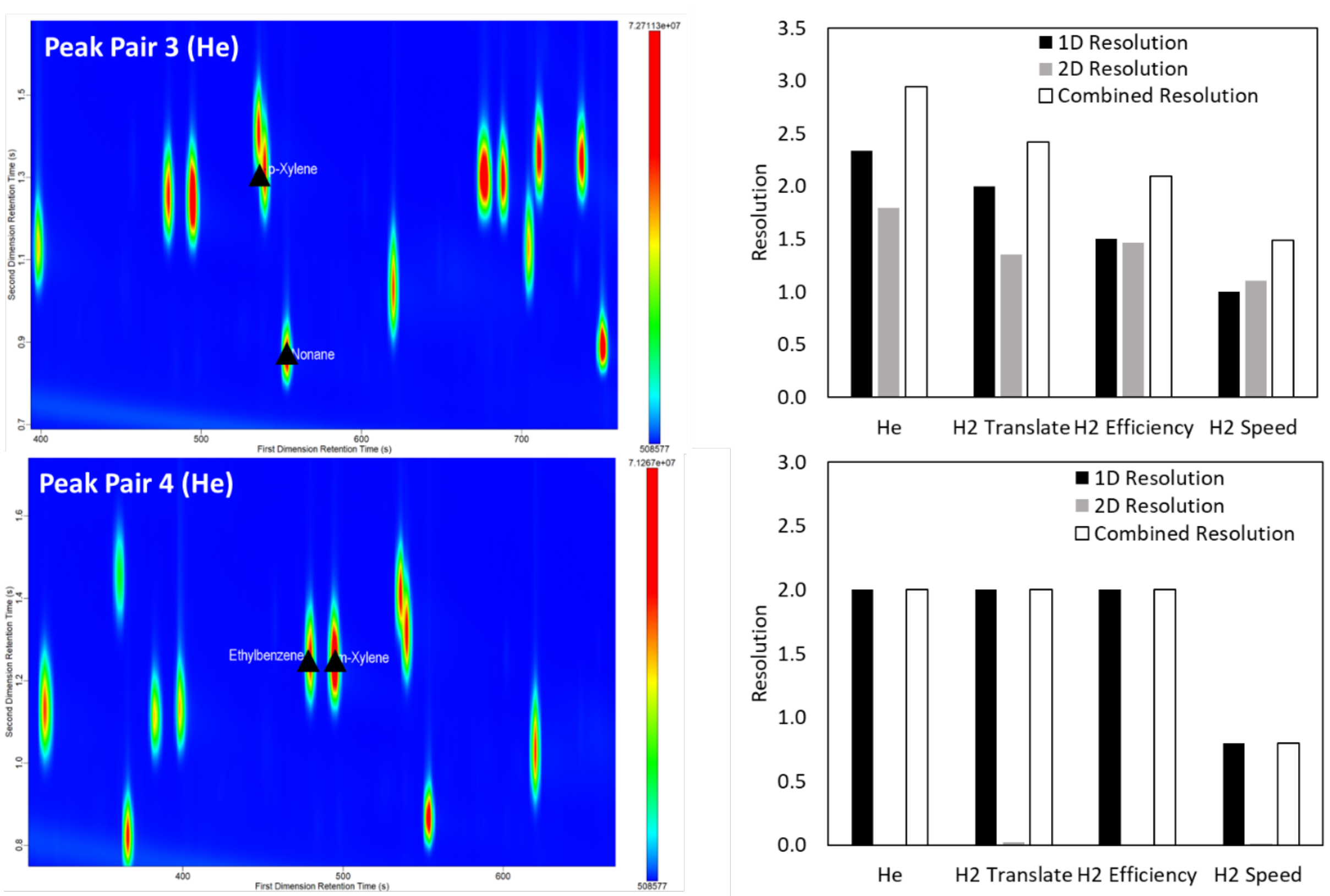

Figure S2. Peak resolution results in first dimension (1D), second dimension (2D) and combined when comparing two examples of peak pairs. Peak pair 3 shows styrene and nonane while peak pair 4 shows ethylbenzene and p-xylene.

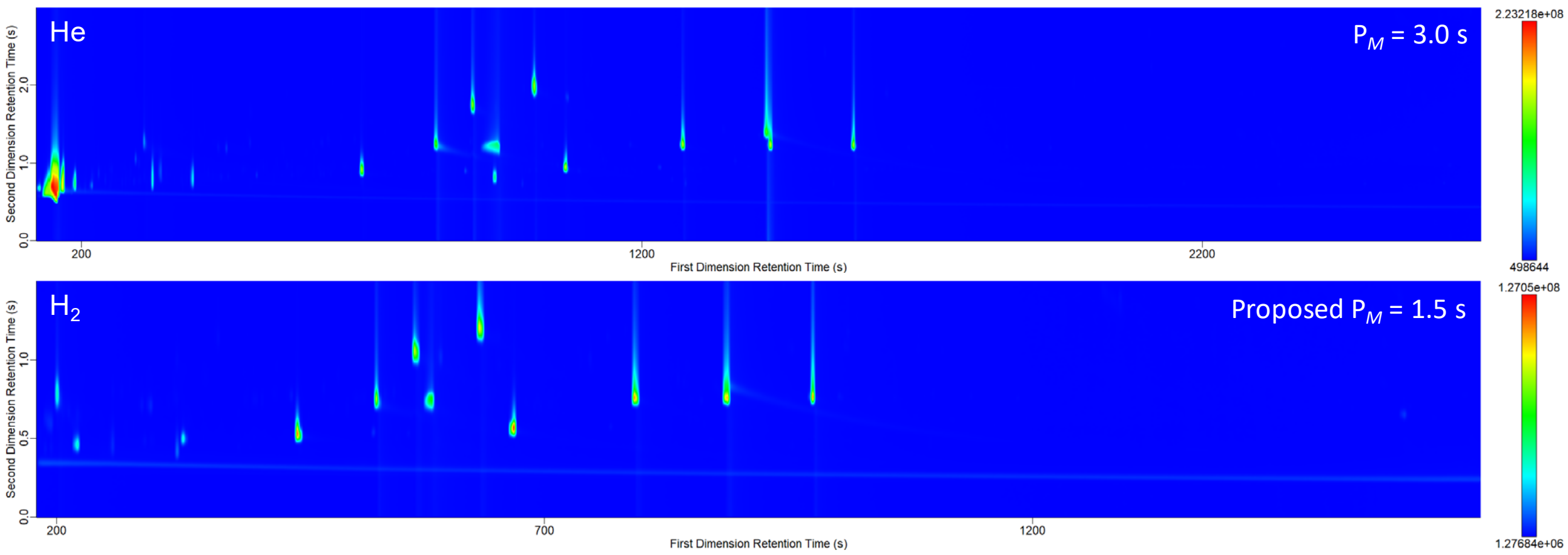

Figure S3. Grob standard contour plots ran on helium (top) and hydrogen (bottom) showing modulation period used and proposed modulation period for a hydrogen translation. The proposed modulation period is shown on the y-axis on the chromatogram.

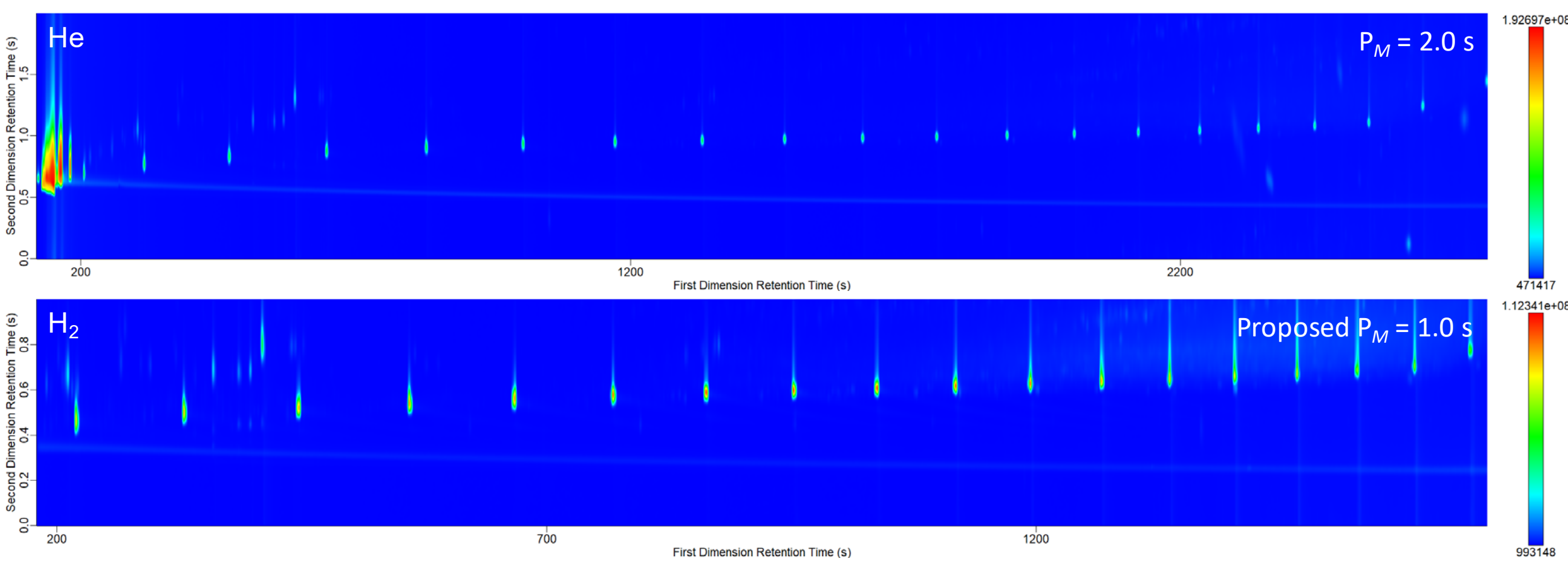

Figure S4. Alkane standard contour plots ran on helium (top) and hydrogen (bottom) showing proposed modulation period for a hydrogen translation. The proposed modulation period is shown on the y-axis on the chromatogram.

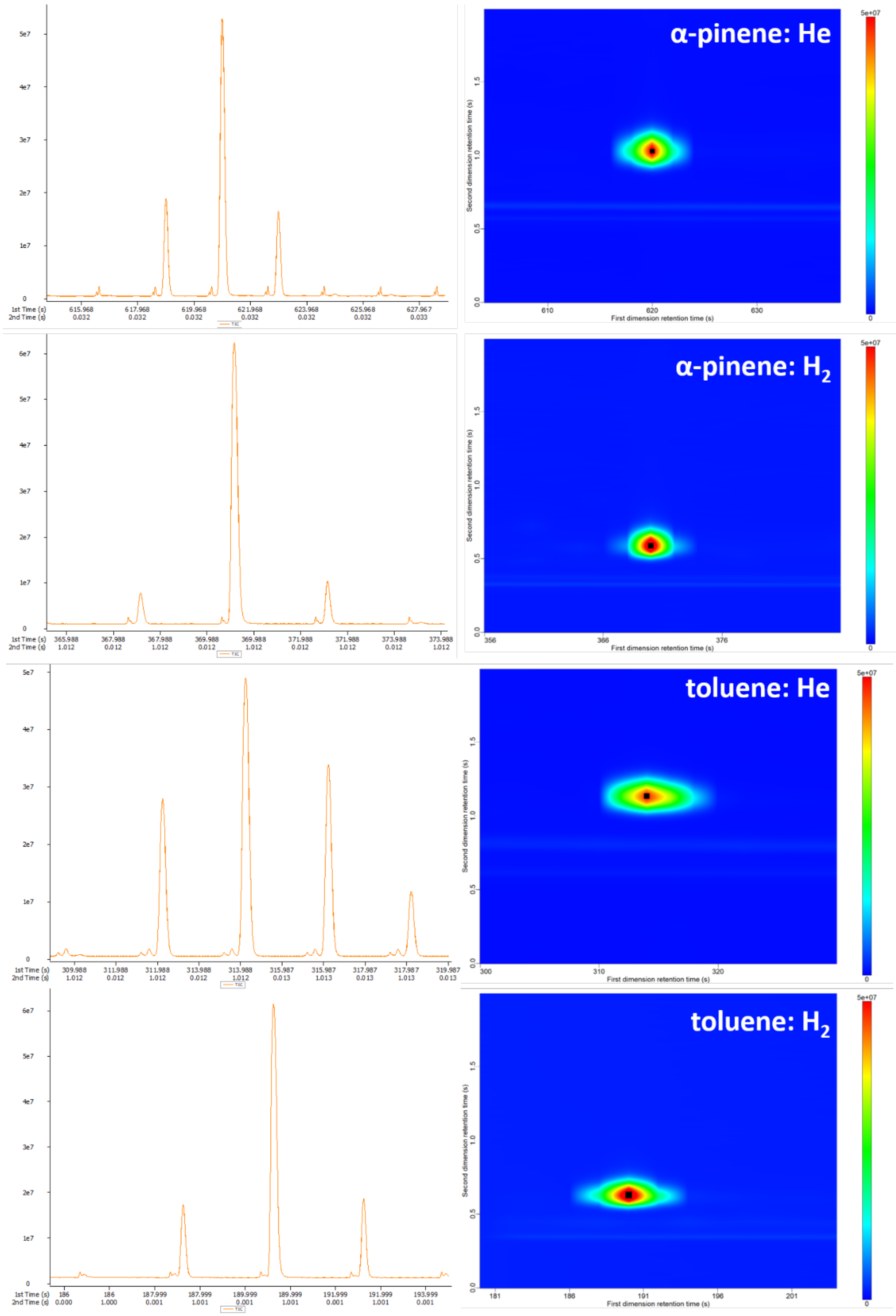

Figure S5. Modulated subpeaks for α-pinene and toluene shown as a 1D chromatogram and corresponding contour plots for each peak

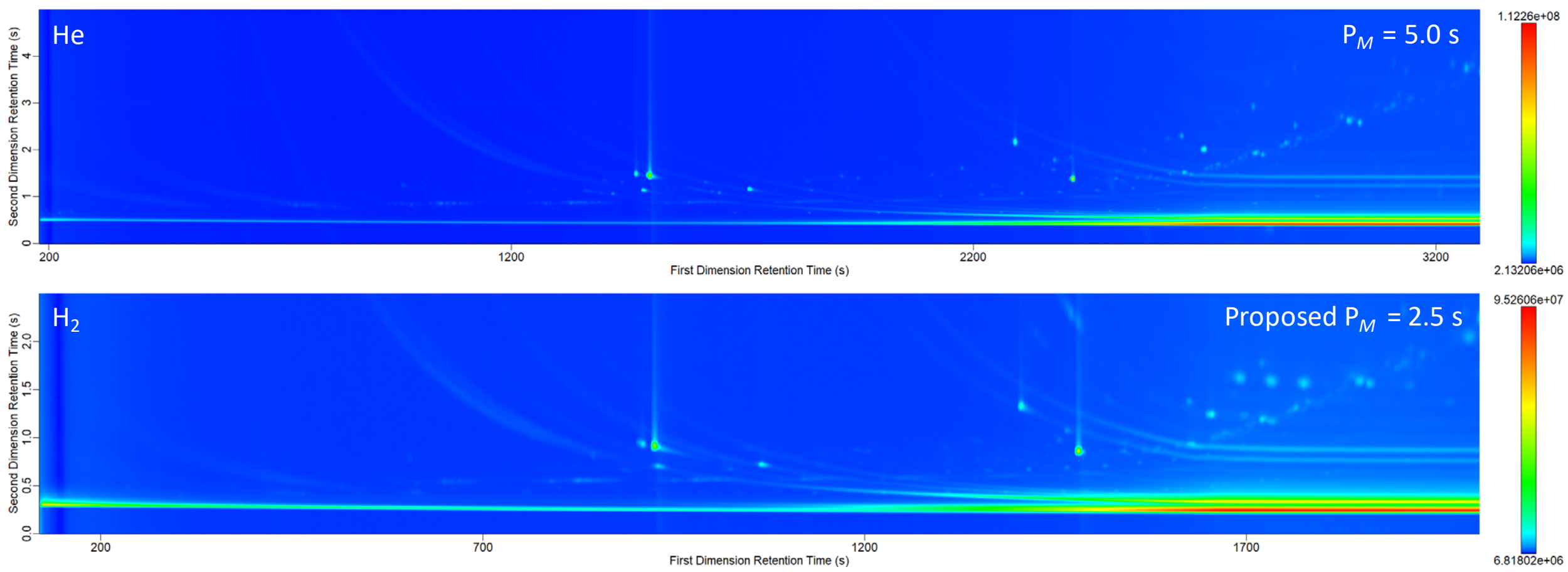

Figure S6. Fingerprint sample contour plots ran on helium (top) and hydrogen (bottom) showing proposed modulation period for a hydrogen translation. The proposed modulation period is shown on the y-axis on the chromatogram. The chromatograms are zoomed in to show the latter half of the chromatogram because analytes are much heavier than the analytes in the other standards.
